# Epigenetic Mechanism and Therapeutic Implications of Atrial Fibrillation

**DOI:** 10.3389/fcvm.2021.763824

**Published:** 2022-01-21

**Authors:** Dan Li, Jiali Nie, Yu Han, Li Ni

**Affiliations:** Division of Cardiology, Department of Internal Medicine and Hubei Key Laboratory of Genetics and Molecular Mechanisms of Cardiological Disorders, Tongji Medical College, Tongji Hospital, Huazhong University of Science and Technology, Wuhan, China

**Keywords:** atrial fibrillation, epigenetic regulation, DNA methylation, histone modification, non-coding RNAs

## Abstract

Atrial fibrillation (AF) is the most common arrhythmia attacking 1. 5–2.0% of general population worldwide. It has a significant impact on morbidity and mortality globally and its prevalence increases exponentially with age. Therapies like catheter ablation or conventional antiarrhythmic drugs have not provided effective solution to the recurrence for AF over the past decades. Over 100 genetic loci have been discovered to be associated with AF by Genome-wide association studies (GWAS) but none has led to a therapy. Recently potential involvement of epigenetics (DNA methylation, histone modification, and non-coding RNAs) in the initiation and maintenance of AF has partly emerged as proof-of-concept in the mechanism and management of AF. Here we reviewed the epigenetic features involved in AF pathophysiology and provided an update of their implications in AF therapy.

## Introduction

The most common arrhythmia is atrial fibrillation (AF). It has an estimated worldwide prevalence of 1.5–2.0% in the general population (1). Currently, over 33 million individuals are suffering from AF worldwide, and the prevalence is anticipated to more than double in the next 40 years (2). AF has substantially increased hospitalization rates, stroke occurrence, and social medical burden and impaired quality of life.

There is a growing understanding of the mechanisms underlying the onset and maintenance of AF. The pathophysiology of atrial fibrillation focuses on ectopic firing promotion and reentrant mechanisms, including ion channel dysfunction, Ca^2+^-signaling abnormalities, structural remodeling, and autonomic neural dysregulation (3). The potential implications of the pathophysiology of AF for its management have been improved rhythm control pharmacotherapy, rate control therapy, AF ablation, and the prevention of thromboembolic events (4). But these treatments have shown limited improvement in AF patients.

**Figure 1:**
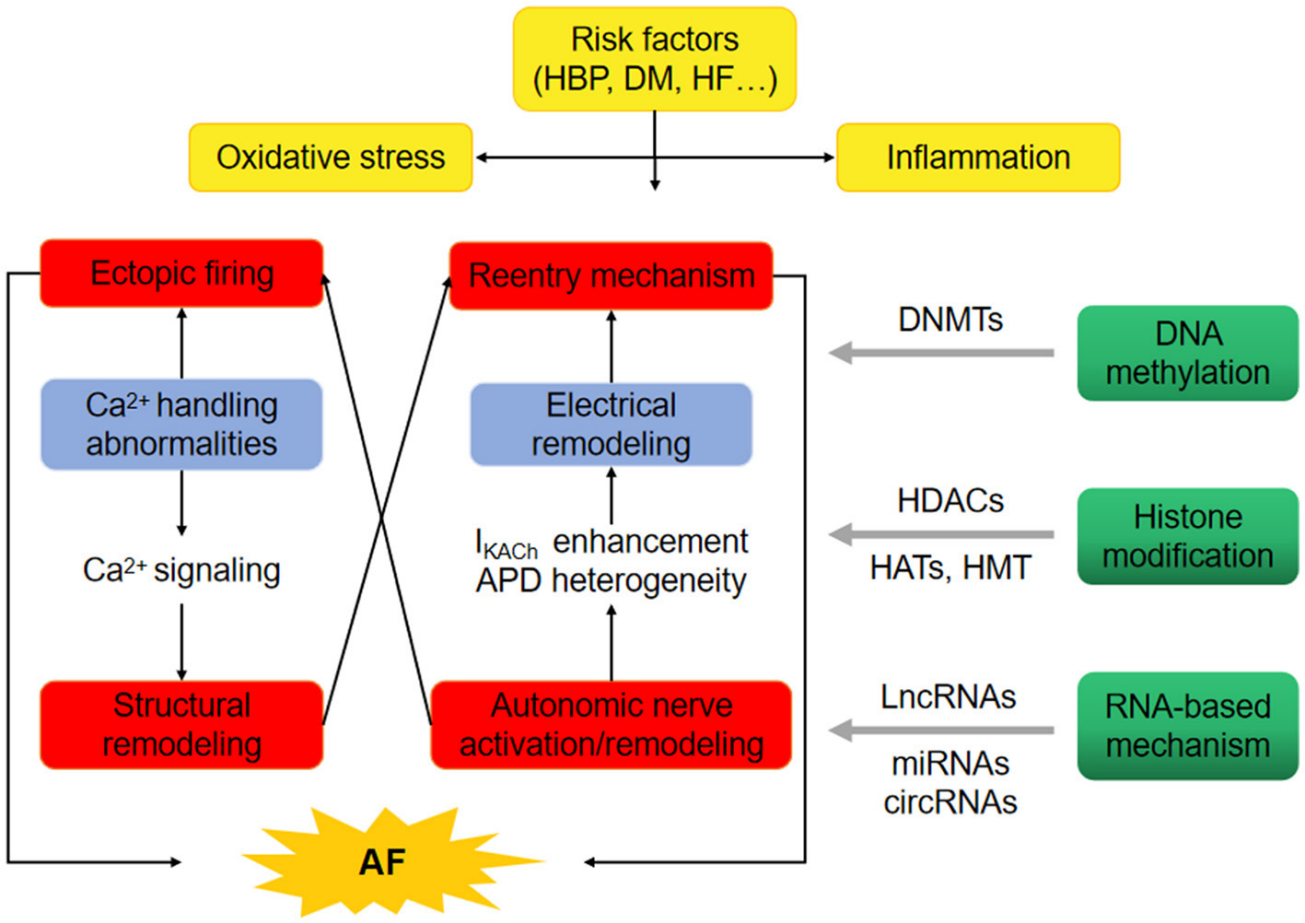
Epigenetics provide the basis for uncovering the pathophysiology of atrial fibrillation (AF). The oxidative stress and inflammation in high blood pressure (HBP), diabetes mellitus (DM) and heart failure (HF) probably promote the occurrence of AF through ectopic firing and reentry mechanism. Epigenetic regulatory mechanisms, involving DNA methylation through DNA methyltransferases (DNMTs), histone modification through histone acetyltransferases (HATs), histone methyltransferases (HMT) and/or histone deacetylases (HDACs), non-coding RNAs such as long-ncRNA (LncRNAs), microRNAs (miRNAs) and circular RNAs (circRNAs) may comtribute to the initiation of AF.

Genome-wide association studies (GWAS) have uncovered over 100 AF associated genetic loci (5). But none has been identified to be a potential therapeutic target, indicating additional candidates to AF pathophysiology. High blood pressure (HBP), diabetes mellitus (DM), and heart failure (HF) are the common risk factors of AF. The oxidative stress and inflammation in HBP, DM, or HF probably contribute to the initiation of AF through calcium signaling related-structural remodeing, electrical reentry mechanism, and autonomic nerve activation. Recently, the possible mechanism linking epigenetics and AF are reactive oxygen species (ROS) levels. One study found that ROS levels promotes AF via increased intracellular Ca^2+^ release by oxidized RyR2 (6). A recent research found that long non-coding RNA ZNF593-AS facilitated RyR2 mRNA stability (7). The changes in epigenetic state induced by ROS levels could provide the basis for uncovering the pathophysiology and management of AF. The functional characterization of regulatory mechanisms, involving DNA methylation, histone modification, and non-coding RNAs, may be related to AF epigenetics (Figure 1) (8). Here we will review the epigenetic features involved in AF pathophysiology and provide a latest functional implications in AF therapy.

## AF Associated Epigenetic Regulation

AF is a highly heterogeneous genetic disease, which consists of distinct subtypes characterized by a few specific chromosomal abnormalities or gene mutations. Although candidate genes and GWAS have identified a number of genetic variants of AF, and elucidated the heritability of AF, a large proportion of AF cases cannot be interpreted by genetic variants alone. Epigenetic regulation including DNA methylation, histone modification, and non-coding RNAs has been explored in the patients and animal/cell modes of AF (Tables 1, 2).

**Table 1:** Epigenetic modification in AF.

**Species**	**Model/tissue**	**Discovery**	**References**
**DNA methylation**			
Human	AF/peripheral blood	2 CpG site associated with prevalent AF (*n* = 183) 5 CpG site associated with incident AF (*n* = 220)	(11)
Human	PeAF/LA	417 differentially methylated CpG sites AF susceptible loci hypermethylation	(12)
Human	valvular AF/RA	DNMT3B → NPR-A promoter hypermethylation	(13)
Rats	ISO treatment/primary cell culture from SD rats	DNMT3A → RAASSF1A-ERK1/2 → cardiac fibrosis	(14)
Human Rats Cell line	VHD/LA ISO treatment/SHR AngII/HL-1 cell	HF → Pitx2 promoter hypermethylation	(15, 17)
Mice Cell line	C57BI6/J mice HL-1 atrial CM	SUR2 promoter hypermethylation	(16)
**Histone modifications and chromatin remodeling**			
Human	AF/Heart		(20)
Human	PeAF/RA, AFB	Elevated expression of EZH2, an HMT specific for H3K27me	(21)
Human Cell line	PeAF/RAA,LAA HL-1 atrial CM	HDAC5 activation and subsequent MEF2-related fetal gene expression	(22)
Human Canine Cell line	PeAF,PAF/RAA,LAA HL-1 atrial CM	HDAC6 activation and subsequent derailment of alpha-tubulin proteostasis and disruption of the cardiomyocyte microtubule structure	(23)
Mice	HopX^Tg^/heart	HDAC inhibition reverses myocardial fibrosis and reduces atrial arrhythmia independent of angiotensin	(24)
Rabbits	PVs and SANs	HDAC6,HDAC8 (≥70% inhibition) HDAC4,HDAC5,HDAC7,HDAC9 (≤50% inhibition) Reduces PVs arrhythmogenesis through calcium regulation	(25)
Human Mice Canine	Sustained AF/HopX^Tg^ Atrial tachypacing dogs	Class I HDAC (HDAC1, HDAC2, HDAC3, HDAC8) inhibition Reduce the total time of fibrillation, atrial fibrosis, intra-atrial adipocytes and immune cell infiltration	(26)
Mice	CREM-IbΔC-X^Tg^	HDAC inhibitor VPA Attenuates atrial remodeling and delays the onset of AF	(31)
**Non-coding RNAs**			
Canine	RAP/atrium	miR-133, miR-590 downregulation → atrial fibrosis	(34)
Human Mice	PeAF/LAA	miR-21 upregulation → atrial fibrosis	(35)
Human Canine Mice	Persistent AF/ Blood,RAA	miR-29b downregulation → ECM protein → atrial fibrosis	(37)
Human	Persistent AF/ LA	miR-1 downregulation → inward rectifier potassium currents upregulation → AF	(39)
Human Canine Mice	AF/RAA	miR-26 downregulation → inward rectifier potassium currents(*I*_K1_) → KIR2.1/KCNJ2 → fibroblast remodeling	(40)
Rabit	Atrial tachypacing model/atrial myocyte	miR-1 → KCNE1,KCNB2 → AERP shortening	(41)
Human Mice	PAF/RAA	miR-106b-25 cluster → RyR2 → calcium leak	(43)
Human Canine	AF/ atrium, LA	miR-223, miR-328, miR-664 upregulation miR-101, miR-320, miR-499 downregulation	(44)
Mice	heart	miR-17-92 and miR-106b-25 downregulation	(45)
Rabbits	AF/atrium	LncRNA(TCONS-00106987) upregulation By sponging miR-26 in electrical remodeling	(47)
Rabbits	AF/RA	TCONS_00075467 sponging miR-328 → electrical remodeling	(48)
Human	AF/atrium	NEAT1-miR320-NPAS2 axis	(49)
Human	AF/EAT	lncRNA differentially expressed in AF and SR	(50, 51)
Human	VHD(50%PeAF)/LAA	circRNA-miRNA-mRNA network DEcircRNA between AF group and SR group (circ 255-ITGA7, circ 418-KCNN2, circ 13913-MIB1, circ 44670-BARD1, circ 44782-LAMA2, circ 81906-RYR2, circ 35880-ANO5, circ 22249-TNNI3K, circ 3136-TNNI3K, circ 56186-TNNI3K)	(52)
Canine	RAP	circRNA-miRNA interaction	(53)
Human	AF/Heart	circRNA-miRNA interaction (e.g., has_circRNA_100612 and has_miR_133b)	(54)
Human	VHD/AA	147 DEcircRNA between AF group and SR group circRNA-miRNA interaction (e.g., has-miRNA-2215p and has_circRNA_0005643) (e.g., has-miRNA-2215p and has_circRNA_0077334)	(55)
Human	AF/peripheral blood	DEcircRNA-DEmiRNA-DEmRNA network (e.g., hsa-circRNA-100053-hsa-mirR-455-5p-TRPV1) (e.g., hsa-circRNA-005843-hsa-mirR-188-5p-SPON1)	(56, 57)
Human	AF/LA	circRNAs sponging activities in PeAF	(59)
Human	RNA sequencing data	Dysregulation of circRNAs in inflammation response in AF	(60, 61)

*AF, atrial fibrillation; PAF, paroxysmal AF; PeAF, permanent AF/persistent AF; LA, left atrium; VHD, valvular heart disease; RA, right atrium; DNMTs, DNA methyltransferases; NPR-A, natriuretic peptide receptor-A; ISO, isoproterenol; SHR, spontaneously hypertensive rats; SD, Sprague-Dawley; AngII,Angiotensin II; RAASSF1A, Ras association domain family 1 isoform A; ERK, extracellular regulated protein kinases; Pitx2, paired-like homeodomain 2; CM, cardiomyocytes; SUR, sulfonylurea recepor; AFB, atrial fibroblast; EZH2, zeste homolog2; HMT, histone methyltransferase; RAA, right atrial appendage; LAA, left atrial appendage; HDAC, histone deacetylase; VPA, Valproic Acid; ECM, extracellular matrix; MEF2, myocyte enhancer factor-2; AERP, atrial effective refractory period; RyR2, ryanodine receptor; HopX, homeo-domain-only protein; PV, pulmonary vein; SAN, sinoatrial node; RAP, rapid atrial pacing; NEAT1, Nuclear-Enriched Abundant Transcript 1; EAT, epicardial adipose tissue; SR, sinus rhythm; VHD, valvular heart diseases; DEcircRNA, differentially expressed circRNA*.

**Table 2:** Target of epigenetic modification in AF.

**Epigenetic modification**	**Target**	**Biological function**	**Ref**
**DNA methylation**			
Hypomethylation	BMP6, BMP8B	TGF-β signaling pathway	(12)
	MSRA, CLU, DUOX2	Response to oxidative stress	(12)
	SLC12A7, SLC38A7, SLC9A9, SCN1A	Sodium ion transport	(12)
	KCNS3, KCNA4, ATP12A, SUR2	Potassium ion transport	(12, 16)
	ITGA5, ITGAE, DOCK1	Integrin-mediated signaling pathway	(12)
	PSMB9, OPRD1, HLA-G, HLA-C, BMP6, HLA-DMB, IL16, MR1, HLA-DRB1, TAPBP, TGFBR3, HLA-DQA1, HLA-DRA	Immune response, Antigen processing and presentation	(12)
	GMCL1L, PPP1R9A, RNASE4, BMP8B, PAX8, CYFIP1, HLX, MGP	Cell differentiation	(12)
	STEAP3, CSRNP1, LGALS7, CLU, DOCK1, UBE4B	Apoptosis	(12)
Hypermethylation	APOL6, OSBPL3, APOA5	Lipid transport, Lipid transport	(12)
	SPDEF, HOXC4, NXN, MSX1, MEIS1, HDAC4, ARHGAP22, DUSP22, HOXA3, EBF4, EBF3, PRM1, DMBX1, PITX2	Development	(12, 15, 17)
	F3, MIB2	Notch signaling pathway	(12)
	NPR-A	Cardiac hypertrophy	(13)
	RASSF1A	Cardiac fibrosis	(14)
**Histone modifications and chromatin remodeling**			
H3K27ac, H3K4me1	GATA4, MYH6, NKX2-5, PITX2, TBX5 CFL2, MYH7, PKP2, RBM20, SGCG, SSPN	Serious heart defects Striated muscle function and integrity	(20)
H3K27me3	EZH2, ACTA2	Atrial fibrosis	(21)
HDAC5 phosphorylation	MEF2-related fetal gene e.g., β-MHC, α-MHC, BNP	cardiomyocyte remodeling	(22)
HDAC6 activation	α-Tubulin	disruption of the cardiomyocyte microtubule structure	(23)
HDAC inhibition	Connexin 40	Atrial structural remodelin	(24)
HDAC inhibition	Ca2+transient amplitudes, sodium-calcium exchanger currents, and ryanodine receptor	Calcium homeostasis	(25)
Class I HDAC inhibition	CD19. CD4, CD163 TNF-α, IL-1β, IL-6, IL-8, leptin, and adiponectin	Angiotensin II signaling in atrial remodeling	(26)
HDAC inhibition	RhoA	Oxidative phosphorylation	(31)
**Non-coding RNAs**			
miR-133,miR-590	TGF-β1, TGF-βRII	Nicotine-induced atrial fibrosis	(34)
miR-21	Spry 1, CTGF	AngII-induced atrial fibrosis	(35)
miR-133, miR30	CTGF	Hypertension-induced LVH TAC-induced LVH	(36)
miR-29b	COL1A1, COL3A1	CHF related-atrial fibrosis	(37, 38)
miR1, miR-26, LncRNA(TCONS-00106987)	KCNJ2	Regulation of K_ir_2.1(subunit of *I*_k1_)	(39, 40, 47)
miR-1	KCNE1, KCNB2	Regulation of subunit of *I*_ks_	(41)
miR-106b-25 cluster	RyR2	calcium leak	(43)
miR-328 TCONS_00075467	CACNA1C, CACNB1	L-type calcium channel regulation	(44, 48)
miR-17-92, miR-106b-25	Shox2, Tbx3	Sinoatrial node dysfunction	(45)
miR320	NPAS2	AngII-induced atrial fibrosis	(49)

*Ref, reference; AF, atrial fibrillation; TGF-β1, transforming growth factor-beta1; TGF-βR1, transforming growth factor-beta R II; NPR-A, natriuretic peptide receptor-A; RAASSF1A, Ras association domain family 1 isoform A; AngII, Angiotensin II; Pitx2, paired-like homeodomain 2; SUR, sulfonylurea recepor; MEF2, myocyte enhancer factor-2; RhoA, Ras homolog gene family, member A; CTGF, connective tissue growth factor; H3K27AC, acetylation of histone H3 lysine 27; H3K4me1, methylation of histone H3 lysine 4; H3K27me3, trimethylation of lysine 27 on histone 3; Spry 1, Sprouty 1; ECM, extracelluar matrix; TAC, transverse aortic constriction; LVH, left ventricular hypertrophy; CHF, congestive heart failure; AngII, angiotensin II; RyR2, ryanodine receptor; NPAS2, neuronal per arnt sim domain protein2*.

### AF Associated DNA Methylation

DNA methylation is well-characterized as a heritable regulation of gene expression. A methyl group is catalyzed by DNA methyltransferases (DNMTs) to shift from the S-adenosyl-L-methionine to the 5' carbon of cytosine which mostly located in cytosine-phosphate-guanine (CpG) islands (9). The gene promoter hypermethylation correlates with transcriptional silencing, whereas hypomethylation leads to increased expression of the gene (10). DNA methylation regulation may serve an important role in AF pathogenesis.

It has been identified the methylation of CpG sites in prevalent, permanent, and paroxysmal AF. Differential methylation of CpG sites were significantly related to prevalent AF (two CpG sites) and incident AF (five other CpGs) by GWAS of the participants' peripheral blood in the Framingham Heart Study (11). The majority of 417 differentially methylated CpG sites discovered in the fibrillating atrium were located in intergenic regions outside of CpG islands (12).

DNMTs dysregulations likely plays an important role in the pathogenesis of AF. Significantly higher level of the whole DNA methylation was found in the AF group than SR group (13). DNMT3b likely contributes to the DNA methylation dysregulations in valvular AF (13). DNMT3A was shown to be involved in Ras association domain family 1 isoform A (RASSF1A)-mediated upregulation of extracellular signal regulated kinases 1/2 (ERK1/2) in cardiac fibrosis (14).

DNA hypermethylation also participated in the association of transcription factor, fibrosis and potassium ion transport with pathophysiology of AF (15, 16). Hypermethylation of paired-like homeodomain 2 (Pitx2) promoter was shown to be associated with AF in humans and aging spontaneously hypertensive rats (SHR) (15). DNA methylation inhibitor 5-Aza-2'-deoxycitidine treatment reduced the left ventricular fibrosis in SHR (13). The CpG hypermethylation of SUR2, a subunit of the ATP-sensitive potassium channel, leads to its silencing expression in the HL-1 atrial cardiomyocyte cell line (16).

Heart failure (HF), a risk factor of AF, may induce Pitx2c promoter hypermethylation *in vivo* and *in vitro* (17) Pitx2c promoter methylation increased and Pitx2c protein level decreased with the increase of DNMT 1 in isoproterenol-induced HF atria compared with normal atria (17). The same trend was shown in Angiotensin II (AngII)-treated HL-1 cells compared with control cells (17). The methylation inhibitor 5-AZa-2 '-deoxycytitine and the AngII receptor blocker Losartan attenuated these effects (17). However, isoproterenol did not alter the expression of Pitx2c and DNMT1 (17).

### AF Associated Histone Modifications and Chromatin Remodeling

Chromatin is the state in which DNA is wrapped in the cell. The nucleosome, the basic unit of chromatin, is an octamer composed of four core histones (H3, H4, H2A, H2B) surrounded by 147 DNA base pairs. A primary component of chromatin that plays an essential role in this regulation is the modification of histones. The core histones are mainly globular except for their unstructured N-terminal “tails.” A distinctive feature of histones, especially their tails, is the large number and type of modified residues they possess.

The gene expression regulation in euchromatin requires the delivery of chromatin-modifying enzymes by DNA-bound transcription factors (TFs). Following the external cues, TFs bind to the specific genes' promoter and promote the gene expression or silencing. So there will be activation-related and repression-related modification for the purposes of transcription. Histone modifications are involved in affecting gene expression. The mono-methylations of H3K27, H3K9, H4K20, H3K79, and H2BK5 are related to gene activation, whereas trimethylations of H3K27, H3K9, and H3K79 are linked to repression.

More and more evidences show that N-terminal tail of histone is subjected to covalent and reversible post-translational modifications, such as methylation and acetylation (18, 19). These modifications are used to modify chromatin compress or create anchoring sites for other transcriptional regulators (18). The methylation and acetylation status of chromatin is regulated by writers and erasers. The most studied representative of writers and erases are histone methyltransferases (HMT) and histone deacetylases (HDACs) (19).

Emerging evidences reveal a role for histone acetylation or methylation to modulate pathogenic gene expression in AF patients. GWAS of AF patients (*n* = 60,620) and controls (*n* = 970,216) indicated active enhancers as indicated by acetylation of histone H3 lysine 27 (H3K27ac) in right atrium, which has demonstrated that AF-associated risk variants fell near the genes acting via structural cardiac remodeling (20). Enhancer of zeste homolog2 (EZH2), a histone-lysine N-methyltransferase enzyme encoded by the EZH2 gene, is participating in histone methylation by binding to H3K27me3 (trimethylation of lysine 27 on histone 3). The expression of EZH2 and H3K27me3 is upregulated in permanent AF patients with atrial fibrosis (21). Compared to the people with sinus rhythm (SR), HDAC3 protein expression and activity levels were increased in paroxysmal AF (PAF), persistent AF (PeAF) (*n* = 5), and long-standing PeAF (*n* = 7) (22). The phosphorylated-HDAC5 levels were correlated with significantly increased BNP gene expression in PeAF patients (22). Similarly, the remarkable elevated expression and activity of HDAC6 were correlated with PeAF duration (23). However, whether the overall acetylation level of protein or the total HDAC activity had no dramatically difference between patients with PeAF/PAF and subjects in SR (23).

Furthermore, the mechanisms in epigenetic regulation of AF, especially HDACs inhibition, have been investigated *in vivo* and *in vitro*. The use of HDAC6 inhibitor tubastatin A *in vivo* can protect dogs with atrial tachycardia pacing from electrical remodeling, while the dominant negative HDAC6 mutant can completely rescue the systolic dysfunction induced by tachycardia pacing (23). HDACs may regulate transcriptional reprogramming in AF. The homeo-domain-only protein (hopx) transgenic mice were recruited into chromatin to induce serum response factor (SRF)—dependent transcription and myocardial hypertrophy. They were administered with or without pan HDAC inhibitor trichostatin A (TSA). TSA treated mice have protective effects on atrial arrhythmia and fibrosis induced by rapid pacing (24). Pan-HDAC inhibitor TSA and class I HDAC inhibitor MPT0E014 reduce the onset of AF by reducing calcium spark through normalize the expression of NCX1 and ryanodine receptors in rabbit pulmonary vein cardiomyocytes (25). Hopx transgenic mice with atrial remodeling and dogs with atrial tachypacing treated with HDAC inhibitor (CI-994) showed no significant effects on cardiac function but decrease of the total time of fibrillation and atrial fibrosis in atrial tachypacing-induced sustained AF (26).

So various HDACs inhibitors are emerging as interesting druggable targets for AF (27, 28). RGFP966, a specific inhibitor of HDAC3, could prevent systolic dysfunction in cardiomyocyte for AF (29). ACY-1215 (ricolinostat), one of HDAC6 inhibitors, will be a potential candidate drug for trials in patients with AF. Because it is undergoing Phase I and II clinical trials for the treatment of multiple myeloma at present with no reports of serious side effects so far (30). Valproic acid (VPA), an HDAC class I/IIA inhibitor, alleviated atrial remodeling in transgenic mice, animal AF models, and human AF (31). And the researchers used ChIP to identify 9 VPA-downregulated genes (Atp5l, Ces1d, Myl7, Ndufa12, Ndufa8, Ndufs7, Pdha1, Tnni3, Uqcr10) (31).

### AF Associated Non-coding RNAs

Most AF-associated GWAS variants reside in the non-coding genome. The non-coding RNAs (ncRNAs) can be classified as short-chain ncRNAs (<200 nucleotides) and long-ncRNA (lncRNAs, >200 nucleotides) (32, 33). The short-chain ncRNAs include microRNAs (miRNAs), transfer RNAs, small nuclearRNAs, small nucleolar RNAs, piwi-interacting RNAs, telomerase RNAs, and other endogenous RNA species (32). CircularRNAs (circRNAs) emerge as novel non-coding RNAs that differ from traditional linear RNAs (33).

The transition from paroxysmal to permanent AF is characterized by a pattern of dysregulated miRNAs, which involve in atrial remodeling or fibrosis, electrical remodeling, calcium signaling, and dysregulation of transcription factors (TFs) (34–42). Mir-133, mir-590, mir-21, miR30, and mir-29b regulated the genes related to arial fibrosis or fibroblast remodeling in atrial fibrillation (34–37). There was upregulation of transforming growth factor (TGF) TGF-β1 and TGFβRII at the protein level and downregulation of miR-133 and miR-590 in the levels of miRNAs in nicotine stimulated atrial fibrosis of AF in dog (34). The angiotensin II (AngII)-induced upregulation of miR-21 and repression of Spry1 was showed in neonatal cardiac fibroblasts (35). Connective tissue growth factor (CTGF) is a secreted protein as a powerful inducer of extracellular matrix (ECM) synthesis. Its levels are substantially increased in hypertension-induced left ventricular hypertrophy (LVH) and transverse aortic constriction (TAC)-induced LVH rodent models (36). The expression of miR29b decreased and the expression of miR29b ECM target-genes collagen-1A1 (COL1A1), collagen-3A1 (COL3A1) increased significantly in congestive heart failure (CHF) atrial fibroblasts (37, 38). The regulation of miR-1, miR-26, miR-208a, miR-328, and miR-499 on genes associated with electrical remodeling promotes reentry circuits by shortening of the action potential duration and effective refractory period (37–40). The increased inward-rectifier K+ current (*I*_K1_), along with increased expression of the principal underlying subunit KCNJ2 mRNA and its encoded K_ir_2.1 protein, has been demonstrate to exhibit a pro-AF-related atrial electrical remodeling. Girmatsion et al. reported that miR-1 levels decreased significantly in human AF and led to increased *I*_K1_ possibly by up-regulating of KCNJ2 (39), miR-26 was found to be downregulated accompanied by upregulation of *I*_K1_/K_ir_2.1 protein in atrial samples of AF patients (40). The slowly activating delayed rectifier potassium currents (*I*_Ks_) and atrial effecitve refractory period (AERP) shortening play an important role in the electronic remodeling of AF. The expression of miR-1 was upregulated to induce AERP shortening by targeting *I*_K1_ channel genes (KCNE1, KCNB2) in right atrial tachypacing in New Zealand white rabbits (41). MicroRNAs have also been shown to control the expression of genes encoding important Ca^2+^ processing and signaling proteins in AF (42). Studies have shown that the atrial level of ryanodine receptor type-2 (RyR2) protein is elevated in paroxysmal AF (pAF) patients, suggesting that post-transcriptional regulation of RyR2 might contribute to the pathogenesis of AF (43). Members of the miR-106b-25 cluster, such as miR-106b and miR-93, could suppress the translation of RyR2 by binding to its 3'-untransted region (UTR) (43). The downregulation of the miR-106b-25 cluster and upregulation of RyR2-mediated sarcoplasmic reticulum Ca^2+^ leak were demonstratedin atria of pAF patients (44). The miR-328 level was elevated by more than 3-fold in AF patients and AF dogs compared to non-AF subjects. Overexpression of miR-328 diminished the L-type Ca^2+^ currents by targeting CACNA1C and CACNB1, which encode its α1c and β1subunits (44). In addtion, miRNA loss-of-function could develop sinoatrial node dysfunction to increase susceptibility of AF (45). miR-17-92 and miR-106b-25 directly supress the genes required for sinoatrial node function, such as Shox2 and Tbx3 (45). Both miR-17-92 and miR-106b-25 inactivation exhibited pacing-induced AF in mice (45).

Several studies have shown that lncRNAs are participanting in basic mechanism of AF development, including electrical remodeling, atrial remodeling, and metabolic remodeling (46). IncRNA TCONS-00106987 was confirmed to promote electrical remodeling of increasing inward-rectifier K^+^ current (*I*_K1_) through endogenous competition with microRNA-26 (miR-26) by luciferase reporter assays and whole-cell patch-clamp recording in AF rabbit model (47). Silencing of TCONS_00075467 can shorten the atrial effective refractory period *in vivo* and reduce duration of the L-type calcium current and action potential *in vitro*, which may also play important roles in electrical remodeling regulation during AF (48). The expression of lncRNAs nuclear-enriched abundant transcript 1 (NEAT1) in atrial tissue of AF patients was up-regulated, and NEAT1 knockdown could improve Ang II-induced atrial fibrosis via the miR-320-NPAS2 axis in mice (49). Recent evidence suggested that lncRNAs in epicardial adipose tissue (EAT) may modulate atrial remodeling (50, 51). Seventeen upregulated lncRNAs and 40 downregulated lncRNAs were differentially expressed in EAT samples collected from persistent non-valvular AF and sinus rhythm (SR) (*P* < 0.05; fold change>1.5) (50). These differentially expressed lncRNAs were mainly related with stress response and metabolic remodeling, which indicating pathogenesis of AF (50). The biological function predictions for the RNA sequencing data collecting from atrial EAT samples of AF and SR revealed that TNF signaling pathway was the most frequent pathway that the lncRNAs might involve in (51).

Recently, more attention has been paid on circRNAs and their association with miRNA and lncRNAs in AF intiation and perpetuation. The differentially expression of circRNAs (Table 1) were verified between persistent AF patients and SR people (52). One hundred and forty-six different circRNAs were found between control and rapid atrial pacing (RAP) dogs (53). And analysis showed that the differentially expressed circRNAs may be involved in the process of “cytoskeleton structural composition and ion channel activity” as well as extensive interaction among different circRNAs and AF related miRNAs and mRNAs (53–55). Integrated analysis speculated that circRNA-microRNA interaction pairs and intricate cross-talk may be involved in AF (Table 1) (56, 57).

Circular RNAs show higher stability than other RNAs. They also exhibit more functional patterns, such as sponging microRNAs (58). The sponging activities of the circRNAs could be responsible for the down-regulation of specific miRNAs in establishment of a permanent AF condition (59).

In addtion, the dysregulated circRNAs may be enriched in participation of inflammatory response in AF (60, 61). 250 up- and 126 down-regulated circRNAs were differentially expressed between AF subjects and healthy donors. It should be noted that the enrichment analysis identified five circRNAs showing the highest significance. Among them, four were enriched in cytokine-cytokine receptor interaction (60). A well-recognized participant for atrial fibrosis association with AF was TGF-beta signaling pathway (61). Differential expression of 14,215 circRNAs were detected in AF patients and healthy controls. Among them, hsa_circ_0000075 and hsa_circ_0082096 was exhibited to be invovled in TGF-beta signaling pathway of the AF pathogenesis (61).

All these researches make ncRNAs promising candidates for the development of latent diagnostic or prognostic biomarkers and even therapeutic targets for AF.

## Epigenetic Modifications in Paroxysmal, Persistent, and Permanent AF

Although differential methylation of CpG sites was found between persistent AF (PeAF) and paroxysmal AF (PAF) in the Framingham Heart study (11), there was no significant difference of the total level of acetylation or the HDAC activity between PeAF and PAF (23). Previous study, whether Genome-wide DNA methylation profiling, HDAC inhibition or role of miR-21, had been performed in permant AF or sustained AF (12, 26, 35). Recent research has been more and more focused on AF promotion (22, 59). Converse role of class I and IIa HDACs was showed in the progression of AF (22). The down-regulation miRNA sponged by circRNA is a characteristics in the transition from PAF to PeAF (59).

## Chromatin Immunoprecipitation (ChIP) and ChIP Sequencing (ChIP-Seq) Study in AF

Chromatin immunoprecipitation (ChIP) is a powful tool to analyze protein-DNA interactions *in vivo*. ChIP-seq, which combines ChIP with second-generation sequencing technology, can efficiently detect genome-wide DNA segments that interact with histones or transcription factors. A ChIP assay showed increased specific binding of EZH2 protein to the DNA of α-SMA promoter, indicating EZH2 was responsible for the atrial fibroblast activation through α-SMA (21). Valproic acid (VPA), an HDAC class I/IIA inhibitor, alleviated atrial remodeling in transgenic mice, animal AF models, and human AF (31). The researchers used ChIP to identify 9 VPA-downregulated genes (Atp5l, Ces1d, Myl7, Ndufa12, Ndufa8, Ndufs7, Pdha1, Tnni3, Uqcr10) (31). miR-26 was found to be downregulated accompanied by upregulation of *I*_K1_/K_ir_2.1 protein in atrial samples of AF patients (40). The ChIP results displayed the binding of nuclear factor of activated T cells (NFAT) to the 3 cis-acting elements in the 5' flanking regions of miR-26 (40). ChIP-Seq analysis revealed that Pitx2 directly bound to conserved chromatin upstream of miR-17-92 and miR-106b-25 (45). All these ChIP and ChIP-seq results suggested the possible signaling pathway in the pathogenesis of epigenetics of AF.

## Epigenetic Therapeutic Implications in AF

Therapies like radiofrequency ablation or pharmacotherapy targeting dysfunction of ectopic firing promotion and reentrant mechanisms have not provided effective solution to the recurrence for AF over the past decades (3–6). RNA-therapy has been paved the way for the translation of experimental studies to human clinical trial. Antisense oligonucleotides (ASO), small interfering RNAs (siRNAs), and microRNAs could consist a potential target in RNA-therapy. However, the most challenging setback is off-target effects. ISIS-CRPRx, a second generation ASO complementary to the coding region of the human c-reactive protein (CRP) mRNA, could reduce the CRP levels substantially but not AF burden in a phase 2 clinical trial in patients with PAF (62). The identification of the epigenetic mechanism and proof-of-concept in preclinical models of AF suggest epigenetic therapies, the so-called epidrugs, will be potential gene therapy approaches for AF.

The epidrugs target and reprogram the epigenome. First- and second-generation epidrugs are small-molecule inhibitors of DNMTs or histone-modifying enzyme. Transcriptional procedures that inhibit DNMTs to reverse DNA hypermethylation and restore sinus rhythm have been proposed as a therapy in AF animals (14, 15). The chronic administration of decitabine (one of the inhibitors of DNMTs) improved ECG arrhythmias and reduced left ventricular fibrosis in a SHR model of AF (15). Although there is poor exploration of inhibitory effect of histone modifying enzymes on AF, they have attracted attention as potential targets for AF therapeutic strategy. Cardiac hypertrophy was reversed by simultaneous treatment of HDAC inhibitors (valproic acid, trichostatin A, and SK-7041) in rodents (63). Administration with tubastatin A (an isoform selective inhibitor of HDAC6) to atrial tachypaced dogs can protects them from atrial remodeling (23). CI-994, second-generation HDAC inhibitors, did not have significant effects on cardiac function in sustained AF model of canine, while the time of fibrillation and atrial fibrosis was reduced (26).

Epigenome plays an important role in regulating cardiac fibroblast activation based on cardiac inflammation, indicating that epidrugs can provide an innovative method to reverse AF related atrial fibrosis (64). EZH2 inhibitor GSK126 can inhibit Ang II induced atrial fibroblast differentiation and ECM production in a mouse model (21).

The epidrugs have been approved for clinical treatment of cancer. Notable examples are the inhibitors of DNMT and HDAC for hematological cancer (65, 66). Azacytidine and decitabine, the first generation of DNMT inhibitors, have been efficacious with poor bioavailability, toxic side effects, and lack of locus-specificity. Second-generation DNMT inhibitors, such as zebularine and guadecitabine, have been developed to make up for the deficiency (65). Some HDAC inhibitors have been approved by FDA for the therapy of hematological malignancies—Romidepsin or Vorinostat for cutaneous T cell lymphoma, Panobinostat for multiple myeloma, and Belinostat for peripheral T cell lymphoma (66). While some HDAC inhibitors are still in clinical trials: Givinostat for haemological/muscular cancer, Entinostat for breast/ovarial cancer, and Resminostat for hepatocellular/colorectal carcinoma. BET inhibitors and HMT inhibitors emerged as the latest generation of epidrugs for cancer, such as OTX-015 or I-BET762, are now in clinical trials (67). However, due to the inadequacy of selectivity and efficacy, these drugs have not been used in the clinical application of AF.

Recently, proteolysis targeting chimeric (PROTAC) has emerged as a promising and potential epidrug. The PROTACs are heteobifunctional molecules with two ligands and a linker: a ligand for binding protein of interest, a ligand for recruiting E3 ligase and a liner connecting the two (68). It can promote the degradation of targeted proteins rather than inhibit the degradation of proteins (68). PROTACs have been used to target specific genetic defects of epigenome modifying enzymes or to develop synthetic lethality in clinic trial of cancer subsets (69). Ongoing studies aimed to selectively use PROTACs to degrade HDAC6 (70). We believe that PROTACs will emerge as a target for AF Implications.

## Conclusions and Future Directions in AF

Epigenetics is a promising and potential tool to reveal unknown mechanisms of AF and for patient-specific pharmacotherapy. Here, we have provided proof-of-concept of epigenetic regulation of histone modifications and non-coding RNA in AF. However, evidences are lacking about the epigenetic regulation of risk factors, such as aging, diabetes mellitus, or ROS, on AF. For example, aging is an important risk factor of AF and aging is associated with global DNA hypomethylation and promoter hypermethylation. But the relationship between aging and DNA methylation in AF remains to be explored.

How to treat AF through correcting associated genetic variants or to modify the epigenome is a challenge and future direction. Recently the emergence of patient-specific human induced pluripotent stem cell-derived CMs (iPSC-CMs) has been as a pharmacologic testing for AF–both for toxicology and efficacy on a patient-specific background (71). CRISPR/Cas9 (epi)genome editing is used in iPSC and animal models to modulate gene function and induce epigenetic changes (72). However, the off-target effect remains these methods to be controversial ethically.

**Figure 2:**
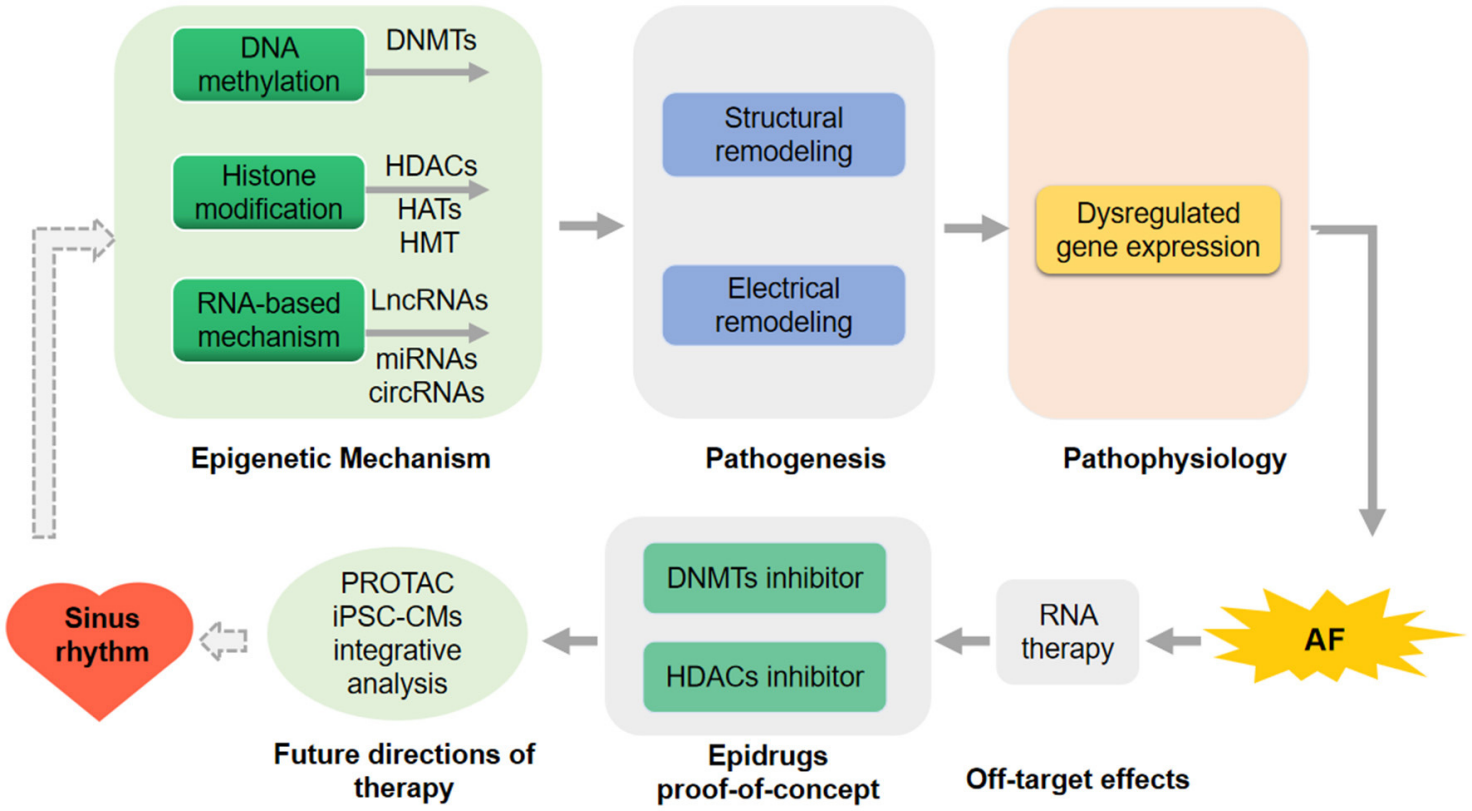
Future direction for atrial fibrillation (AF) prediction and therapeutic intervention. RNA-therapy has not applied in the therapy of human being because of the off-target effects in the human clinical trial. DNA methyltransferases (DNMTs) inhibitors and histone deacetylases (HDACs) inhibitors as the epidrugs have been proof-of-concept in preclinical models of AF. proteolysis targeting chimeric (PROTAC), human induced pluripotent stem cell-derived CMs (iPSC-CMs) and the integrative analysis using computational modeling or machine learning in a patient-specific mode will be performed to further improve strategies for AF intervention.

Integrative analysis using computational model and machine learning to better associate epigenetic changes with AF will bring new insights (73). Performing these analyses (PROTAC, iPSC-CMs, and the integrative analysis using computational modeling and machine learning) in a patient-specific mode will further improve strategies for AF prediction, diagnosis, therapeutic intervention, and prognosis (Figure 2).

## Author Contributions

LN design the study. DL take the responsibility of literature search and manuscript writing. All authors critically revised the manuscript for important intellectual content and gave final approval for the version to be published.

## Funding

This work is supported by National Natural Science Foundation of China (Nos. 82070354 and 81470519) and Program for Huazhong University of Science and Technology Academic Frontier Youth Team (No. 2019QYTD08).

## Conflict of Interest

The authors declare that the research was conducted in the absence of any commercial or financial relationships that could be construed as a potential conflict of interest.

## Publisher's Note

All claims expressed in this article are solely those of the authors and do not necessarily represent those of their affiliated organizations, or those of the publisher, the editors and the reviewers. Any product that may be evaluated in this article, or claim that may be made by its manufacturer, is not guaranteed or endorsed by the publisher.
